# COVID-19 vs. stakeholder engagement: the impact of coronavirus containment measures on stakeholder involvement in European energy research projects

**DOI:** 10.12688/openreseurope.13683.3

**Published:** 2021-10-14

**Authors:** Diana Süsser, Andrzej Ceglarz, Vassilis Stavrakas, Johan Lilliestam

**Affiliations:** 1 Institute for Advanced Sustainability Studies (IASS), Potsdam, Germany; 2 Renewables Grid Initiative, Berlin, Germany; 3 Technical University Munich, Bavarian School of Public Policy, Munich, Germany; 4 University of Piraeus, Technoeconomics of Energy Systems laboratory (TEESlab), Piraeus, Greece; 5 University of Potsdam, Faculty of Economics and Social Sciences, Potsdam, Germany

**Keywords:** COVID-19, coronavirus, stakeholder engagement, transdisciplinarity, energy research, Horizon 2020, EU

## Abstract

The coronavirus (COVID-19) pandemic has affected societies and economies around the world, and the scientific community is no exception. Whereas the importance of stakeholder engagement in research has grown quickly the consequences of the pandemic on this has so far not been empirically studied. In this paper, we investigate the effects of the COVID-19 crisis on European energy research, in particular the stakeholder work, during the first wave of the coronavirus in spring and summer 2020. We pose the research questions: (i) How much of a problem are the coronavirus containment measures for stakeholder engagement? (ii) How have researchers coped with the situation, and (iii) How do they evaluate alternative stakeholder activities implemented? We conducted an online survey among European energy research projects with stakeholder engagement between June and August 2020. We found that only one of six engagement activities could be implemented as planned, whereas almost half were cancelled or delayed. The most common coping strategies were changing involvement formats – mainly to webinars or online workshops – or postponement. Whereas respondents are largely satisfied with one-to-one and unidirectional online formats, such as webinars, online interviews, and online surveys, they see interactive group activities as less suitable for online engagement. Most respondents plan to continue using online formats to complement, but not to replace, physical meetings in future research. All long-term effects remain to be seen, but given the postponement of many stakeholder involvement activities, many projects may face problems at later stages of their realisation. These findings suggest that the pandemic may have catalysed a rapid introduction of specific online formats in academic stakeholder interaction processes.

## Introduction

The coronavirus 2019 (COVID-19) pandemic has strongly affected societies and economies across the world, including the scientific community. The social distancing and lockdown measures applied in most countries have potentially influenced one particular aspect prominent in many research projects: stakeholder involvement. With the growing importance of stakeholder interaction in research, the tie between science and practice has improved, but science has also become vulnerable to the availability and readiness of stakeholders to interact with researchers. Based on our own experiences, we expected that many researchers have faced specific challenges to interact with stakeholders online, and they had to “experiment” with different online formats and tools. Hence, it is important to understand the impacts of the pandemic on stakeholder involvement in research and the satisfaction of coping measures to draw implications on what this new mode of online stakeholder involvement means for future choices of physical and digital stakeholder activities.

In this paper, we investigate the effects of the COVID-19 crisis on the stakeholder work in European energy research (focusing mainly, but not exclusively, on Horizon 2020 projects), during the first wave of the coronavirus pandemic in spring and summer 2020. We address three research questions: (i) how much of a problem are the coronavirus containment measures for stakeholder engagement in European energy research projects?, (ii) how have researchers responsible for stakeholder engagement coped with the new situation?, and (iii) how do researchers evaluate the coping measures (if undertaken)? We report and discuss the findings of a survey distributed to all running European Union (EU) funded energy projects with stakeholder components, carried out in June-August 2020, investigating the effects of coronavirus containment measures on stakeholder involvement in European energy research. This study is not only relevant for the scientific community to gain a better understanding of applied coping strategies for stakeholder engagement in the times of the COVID-19 pandemic, but also for funding bodies, who have to make decisions about how to support research projects under the new conditions.

## Background: stakeholder engagement in research projects and COVID-19

In recent years, the importance of stakeholder involvement and transdisciplinarity in sustainability research has grown quickly, including energy research ( Fazey *et al*., 2018; Lutz & Bergmann, 2018; Mielke *et al*., 2016). Where values are contested ( Funtowicz & Ravetz, 2006), transformations are conflicting ( Renn, 2019) and decisions are urgent, transdisciplinarity is an answer for developing societally relevant solutions to complex, real-world problems ( Lang *et al*., 2012; Lutz & Bergmann, 2018; Scholz & Steiner, 2015). Furthermore, stakeholder involvement can increase the relevance of research, bring higher acceptability and accountability of the problem, and increase legitimacy and societal ownership of the research. Given these potential benefits of engaging stakeholders, funding bodies also now encourage, and oftentimes require, the involvement of stakeholders in research (e.g., European Commission, 2020b).

Stakeholder involvement is today much more than a social-scientific add-on: these engagement activities shape the projects themselves, often including co-creation of both research questions and project aims, and the projects often seek to influence the societal processes with which they engage ( Bracken *et al*., 2015; Klenk *et al*., 2015). Stakeholders can be involved in research to different degrees, encompassing information, consultation, cooperation, collaboration and empowerment ( Schneider & Buser, 2018; Stauffacher *et al*., 2008). Although the degrees of engagement depend on research-project phases ( Bruhn *et al*., 2019) and involvement formats ( Mielke *et al*., 2017), many of them are based on the physical presence of stakeholders in one location – and these have been particularly strongly affected by the COVID-19 crisis.

Academia responded to the coronavirus pandemic and its containment measures in various ways: normatively, by encouraging the promotion of a culture of care and the redefinition of excellence in teaching or research, e.g., by focusing more on inequalities in academic institutional environments ( Corbera *et al*., 2020), but also pragmatically, by quickly adapting to the distancing measures and moving academic interactions, like lectures, seminars and conferences, online ( Leal Filho *et al*., 2020; Schwarz *et al*., 2020). Similarly, funding bodies reacted to the coronavirus outbreak too. For example, the European Commission announced that the “ *force majeure”* clause can be invoked in Horizon 2020 projects, if the grant beneficiaries are not able to fulfil their obligations due to coronavirus restrictions ( European Commission, 2020a), including the stakeholder engagement activities. This is very important, because in more stakeholder-dependent projects, the COVID-19 crisis certainly has the potential to make entire projects unfeasible. Recent research ( Corbera *et al*., 2020; Leal Filho *et al*., 2020) provides important findings on how academia and sustainability researchers have been impacted and have dealt with the crisis, but did not address the impact on stakeholder engagement in research projects. We contribute to the closing of this gap by investigating the impacts on stakeholder engagement in energy research and providing insights into how the research community has coped with the restrictions, as well as what has worked best in the first months after the coronavirus pandemic started.

## Methods

To identify the impact of the COVID-19 crisis and containment measures on stakeholder involvement in energy research projects, we carried out an online survey study among people responsible for stakeholder engagement who work in energy research projects across Europe. The survey was done in summer 2020, around four months after the coronavirus outbreak in Europe. During spring and early summer 2020, all European countries had COVID-19-related containment measures in place. These included lockdowns in most countries – leading to a closure of many academic and research entities, cultural institutions and other public spaces as well as strict restrictions on non-essential travels, and introduction of social distancing rules on meeting other people.

The survey was designed as a collaboration between researchers for the projects SENTINEL (Horizon2020; energy), TRIPOD (European Research Council; energy), and PANDORA (Horizon2020; fisheries) ^1^ as an explorative, semi-quantitative, self-completion online questionnaire (cf. Bryman, 2012), using the online tool “LimeSurvey” ( LimeSurvey, 2020). Survey questions were structured around five blocks:

- A. general questions concerning the stakeholder engagement activities in the projects;
- B. COVID-19 impacts on stakeholder engagement;
- C. coping strategies and alternative formats implemented in response to coronavirus restrictions;
- D. evaluation of the implemented alternative formats; and
- E. demographic data.

The survey contained independent questions as well as questions that built on previous answers. We used different question formats, from Likert-like scales to multiple choice and free text boxes, depending on the variables to be addressed. We pre-tested the survey with our project partners and adapted it in response. The questionnaire is available as extended data ( Süsser *et al.,* 2021).

For data collection, we identified 195 Horizon 2020 energy research projects relevant to our study. The CORDIS database brought 365 hits of projects using the search keywords ‘energy’ and ‘stakeholder’, which started no later than January 2020 and ran at least until the end of 2020. We contacted only 195 out of the 365 projects, as the rest of the projects did not focus on energy questions, were rather technological/industry-focused without a clear stakeholder component, and/or did not provide any contact details. We distributed the survey widely via email to projects identified in the CORDIS research database, and existing networks (e.g. partner projects). In addition, the European Commission Directorate-General of Research & Innovation helped with the distribution of the survey. We also promoted the survey via social media channels, such as ResearchGate, Twitter and LinkedIn, specifying that the survey should be completed only by project coordinators or partners responsible for stakeholder engagement activities. The survey was online for twelve weeks during the period June-August 2020. We allowed also for non-Horizon2020 to participate in our survey, to expand the sample and broaden the respondent base to capture experiences from a higher project diversity. Additionally, we allowed for responses from multiple researchers from one project, because different stakeholder activities are often performed in different temporal and geographical contexts, each with a different containment situation. We treated multiple responses from single projects as individual responses.

We analysed the statistical survey in three steps: first, we compiled and compared the quantitative responses; the resulting descriptive analysis is the core of the results below ( Creswell & Creswell, 2018). Second, we complemented the results based on the written replies. Third, we applied a logistic regression analysis using two different discrete choice models to derive preliminary insights on the correlation of the i) “very negatively” and ii) “not at all” responses in terms of the impact of COVID-19 on stakeholder engagement with relevant explanatory variables from other categories of the survey. We performed a logistic regression analysis to further shed light on potential factors that could explain the very negative impacts of COVID-19 on the stakeholder engagement activities of the energy research projects, but also on factors that could explain the zero effect of the pandemic on stakeholders’ involvement. Our goal was not to perform a complete econometric analysis (e.g., best-fitting model information criteria, evaluation of parameter estimates using quasi standard errors, etc.), but to identify meaningful correlations between the variables under study to complement the explanatory analysis of the descriptive statistics of the survey data.

### Methodological details on the logistic regression analysis

As dependent variables, we selected the two marginal cases *“COVID-19 pandemic influence on stakeholder activity/engagement– Not at all”* and *“COVID-19 pandemic influence on stakeholder activity/engagement– Very negatively”* to help us further derive some meaningful explanations. The latter is also attributed to the fact that, given the format that the online survey took place, our sample did not allow for the development of one theoretical prediction model that could include all the different Likert-scale responses. We converted both cases to two separate dummy variables (“1 = YES to the question”, “0 = NO to the question”). For each one of the two dependent variables, we formulated a separate discrete choice model and selected an initial set of explanatory variables based on its relevance to each one of them. We then converted again the categorical explanatory variables to dummy variables. We made the final selection of the explanatory variables to be included in each discrete choice model through a trial-and-error approach: Different sets of relevant explanatory variables were tested, examining in parallel the correlation matrix to test for collinearity issues among the covariates, until no significant collinearities were observed, to ensure that each final set of explanatory variables is able to adequately predict the respective dependent variable.

For both discrete choice models, the probability that the stakeholder engagement activities of each project *i* are very negatively affected (or are not affected at all) is modelled:

$$P({\text{y}}_{\text{i}}\;\text{=}\;\text{1})\;=\;\Lambda (\text{β}\cdot {\text{x}}_{\text{i}})$$

where:

○ y _i_ is the dependent variable describing if the stakeholder activities of a project i are very negatively affected, or are not affected at all;
○ x _i_ is the vector of independent/explanatory variables for the i ^th^ project;
○ β is the parameter vector to be estimated; and
○ Λ is the logistic distribution ( Spyridaki *et al.*, 2020).

The logistic cumulative distribution function is defined as:

$${\text{P(y}}_{\text{i}}\;\text{=}\;{\text{1|x}}_{\text{i}}\text{)}\;\text{=}\;\text{Λ(β}\cdot {\text{x}}_{\text{i}}\text{)}\;\text{=}\;\frac{{\text{e}}^{\text{β}\cdot {\text{x}}_{\text{i}}}}{\text{1}\;\text{+}\;{\text{e}}^{\text{β}\cdot {\text{x}}_{\text{i}}}\;}$$

where P is the probability of y occurring. The maximum likelihood (ML) estimation method is used to estimate the parameter vector β.

### Sample description

**Figure 1.**
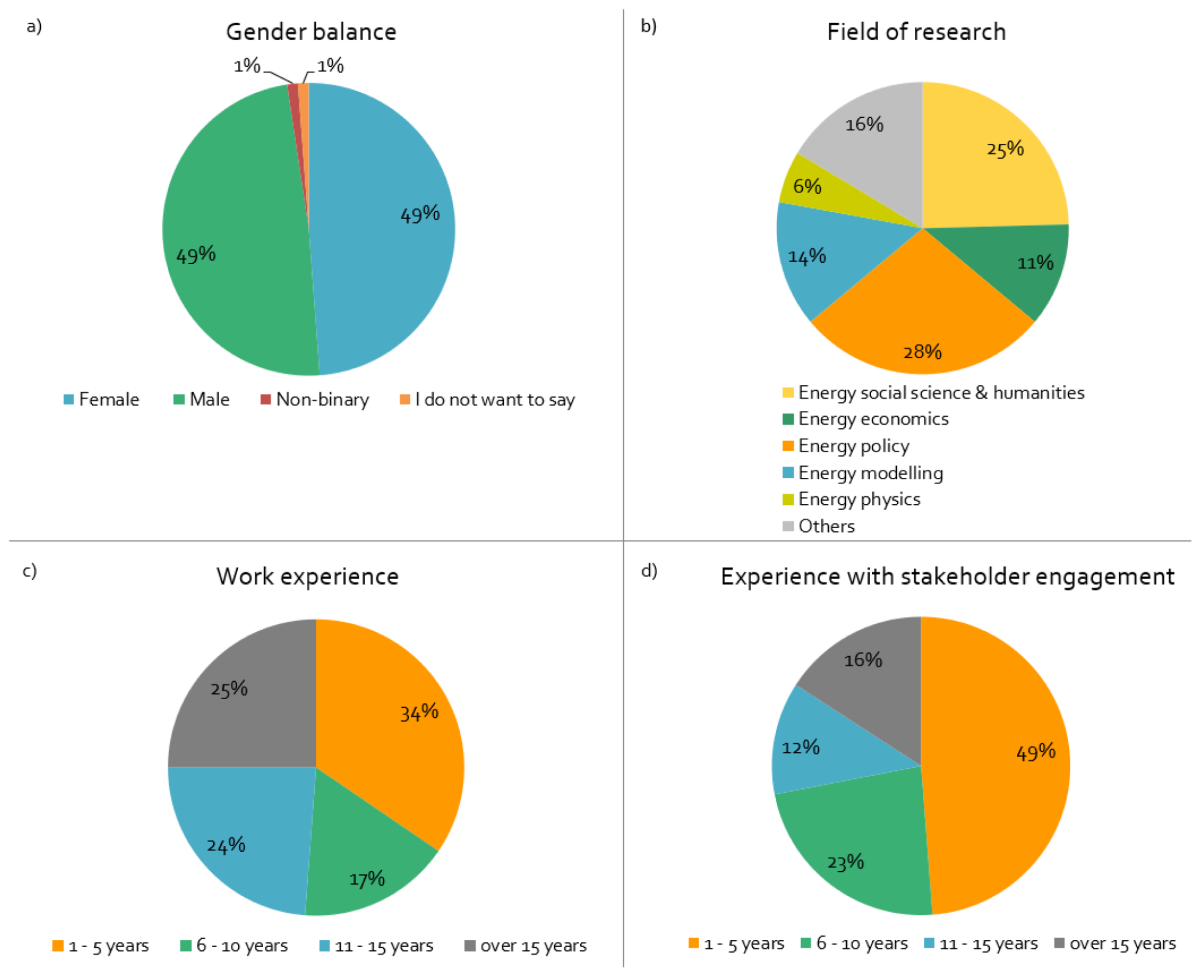
Demographics of surveyed respondents: **a**) *What is your gender?,* **b**) *What is your main field of research?,* **c**) *How long have you worked in this field?,* **d**) *How long have you been engaging with stakeholders in European Union projects?,* n = 84.

We received 84 complete responses from 72 different energy projects: 62 different EU Horizon 2020-funded projects (31% of the stakeholder-engaging EU-funded energy research projects running at the time), and 10 projects with other funding sources. For most projects, we received only one response; for eight projects we received two responses ^2^, and five responses from one project. Most projects started in 2018/19 and will end in 2021/22; practically all are three-year projects. The important demographic sample data of surveyed respondents is summarised in Figure 1.

### Ethics requirements

The research has been conducted under the ethics requirements and guidelines of the SENTINEL project (Deliverables 11.1 and 11.2), which follows the guidelines of the European Commission. We have applied an ethically-robust methodology for the data collection and processing in the context of this study, under the guidance of the Institute for Advanced Sustainability Studies (IASS) data protection service. This has been supported by bilateral data protection agreements. The respondents agreed to our data protection standards via a GDPR disclaimer, and by participating in the survey.

## Results

### Stakeholder engagement in energy research projects

**Figure 2.**
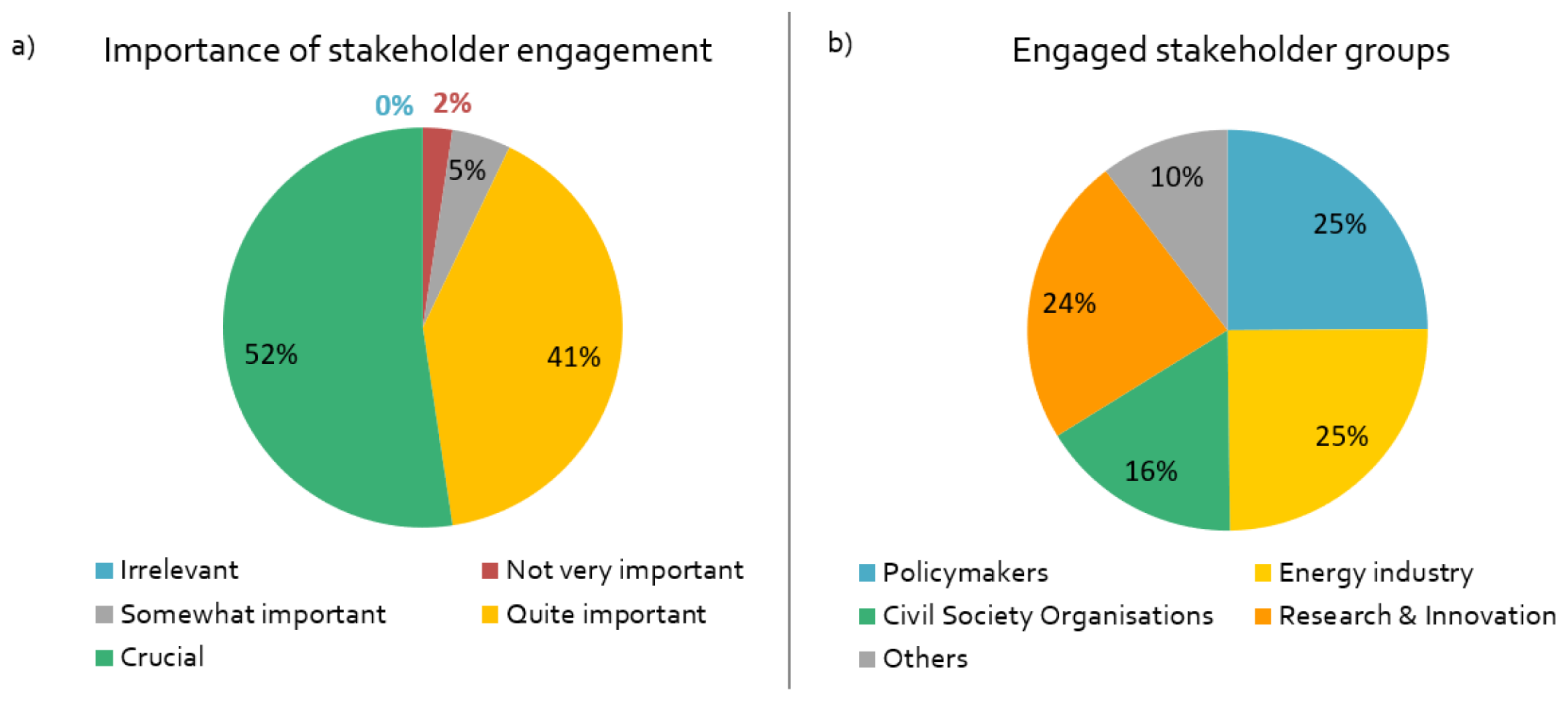
Importance of stakeholder engagement and engaged stakeholder groups: **a**) *How important is stakeholder engagement for the success of your project?,* **b**) *What stakeholder groups are being engaged in your project?,* n = 84.

Stakeholder engagement is a crucial or important component of by far most projects in our sample ( Figure 2a). The researchers mainly engage with stakeholders periodically in specific phases of the project ( Figure 2b). Most of the respondents engage with EU stakeholders; about one third of the projects also work with non-European stakeholders, for example in the US, China or Indonesia.

According to the original project plans, 2020 was supposed to be a major year for stakeholder engagement for almost all respondents; hence almost every project was affected in some way by the coronavirus containment measures in Europe. A variety of physical and online activities were planned in 2020 – mainly workshops, information events and conferences. Almost half of the respondents had planned online interaction formats, such as webinars.

**Figure 3.**
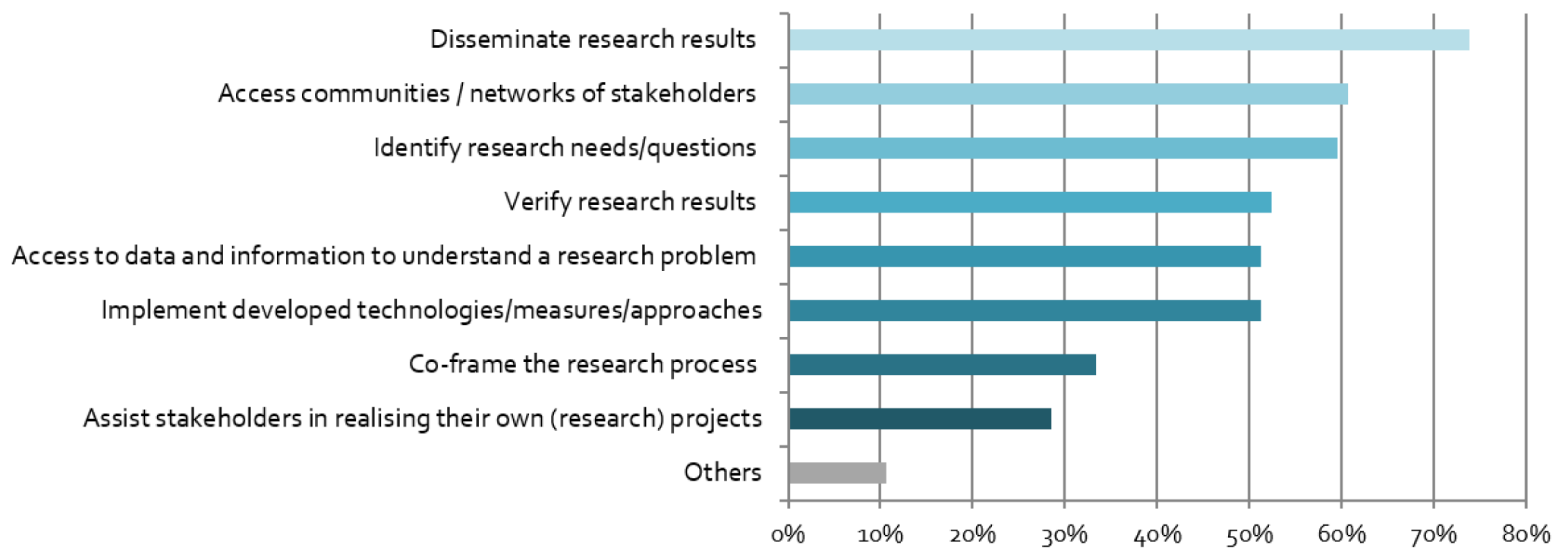
Motives for stakeholder engagement: *What is the purpose of your stakeholder engagement? (multiple responses possible),* n = 84.

The respondents have different motives for engaging with stakeholders. As shown in Figure 3, the linear research mode is dominant, in which stakeholders are either viewed as the target audience for results (“dissemination”), or as research subjects (“access stakeholders”). However, more transdisciplinary and co-creative motives are also high on the agenda, including research question identification and implementation of findings/technologies.

### The impacts of the coronavirus containment measures on stakeholder engagement activities and outcomes

**Figure 4.**
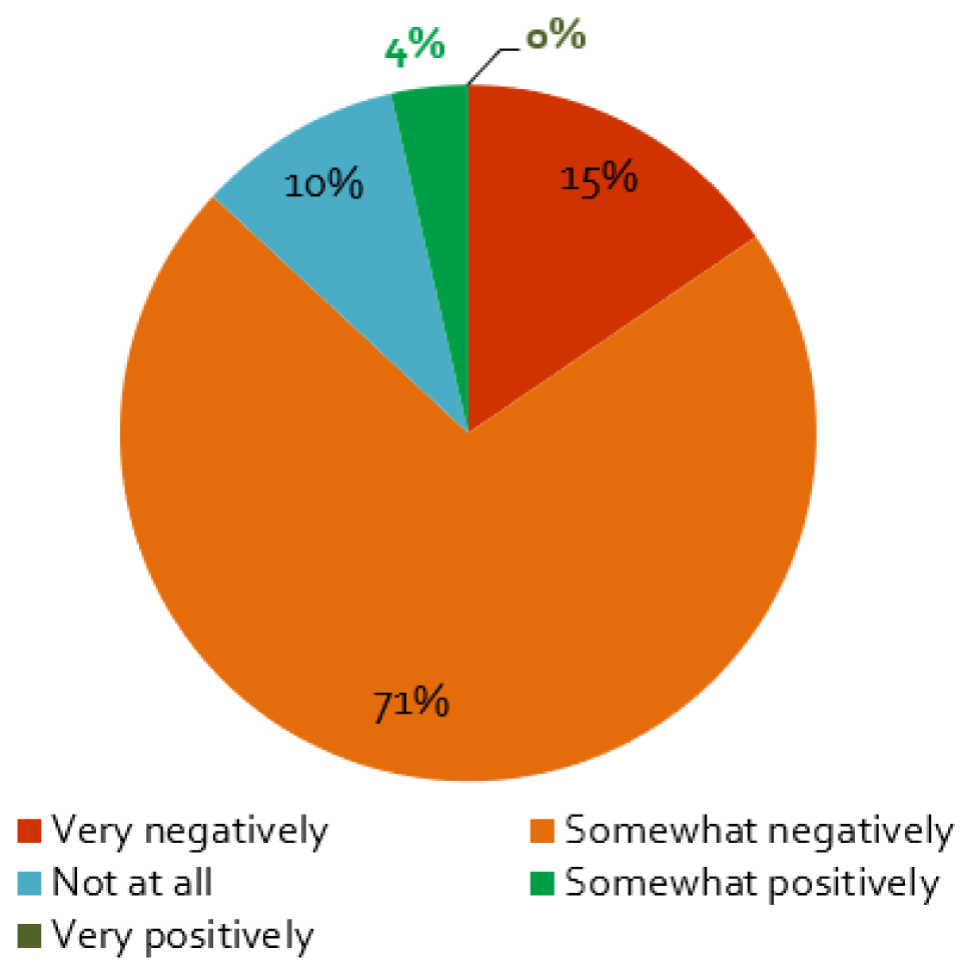
Impact of the coronavirus disease 2019 (COVID-19) pandemic on stakeholder activities: *Does the COVID-19 pandemic influence your stakeholder activity/engagement negatively or positively?*, n = 84.

The first wave of the coronavirus and its containment measures affected stakeholder activities in energy research projects mainly negatively: almost nine of ten respondents perceive somewhat or very negative effects ( Figure 4). Furthermore, projects planning face-to-face workshops in 2020 are more negatively affected by the crisis, which is not surprising, as such events were *de facto* banned in most countries by social distancing measures. The same applies to projects where stakeholder involvement has a higher priority: our regression analysis shows a positive correlation between the projects that stated a very negative influence and responses that stated that stakeholder engagement is crucial for the successful implementation of the project ( Table 1). In addition, projects that focused on engaging with policymakers were more affected: we find a negative correlation between no influence (‘not at all’) and projects engaging with policymakers, but not for those engaging with other stakeholder groups, a negative correlation between negative effects and projects engaging with energy industry representatives, and positive correlation between no influence and projects engaging with civil society organisations. This could be due to the fact that, typically, engagement activities that involve policymakers take place as physical workshops and meetings, while activities that involve energy industry and civil society representatives took place in various formats, including online channels also before the COVID-19 pandemic. Table 2 shows the detailed results of the regression analysis.

**Table 1.** Logistic regression model I. Dependent variable: “Impact of COVID-19 on stakeholder engagement of energy research projects – *Very negatively*”.

Explanatory variables		Prediction model
Category	Name	Coefficient
**Stakeholder groups engaged**	Policymakers	-3.224 (2.333)
	Energy industry	-3.726 (2.076) *
**Geographical/spatial focus**	EU	-11.974 (5.945) **
	COVID-19 cases	7.116 (3.194) **
**Frequency of engagement**	Weekly	-5.075 (4.310)
**Importance of stakeholder engagement for the success of the project**	Crucial	9.043 (4.083) **
**Year of stakeholder engagement according to project plan/proposal**	2021	-9.139 (4.033) **
	2022	5.058 (2.893) *
**Engagement activities according to the project plan/proposal (year 2020)**	Face-to-face workshops	6.999 (3.579) **
	Information events for stakeholders	-6.269 (3.009) **
	Face-to-face interviews	1.920 (1.745)
**Purpose of stakeholder engagement**	Disseminate research results	0.917 (1.895)
**Priority of stakeholder engagement changed due to COVID-19**	Priority decreased	6.768 (3.736) *
**Change in relationship to stakeholders**	Stakeholders priority has shifted away from the project	-3.719 (2.497)
	It is harder to reach stakeholders	4.886 (3.708)
**Impact of changes in your stakeholder engagement activities on proceedings and results of your overall project**	Delays in the flow of data to other work packages	5.366 (2.683) **
	The project duration will need to be extended	6.037 (3.045) **
	Deliverables’ submission has been/will be delayed	3.548 (2.288) *
**Constant**		-2.336 (6.257)

**Notes:**       -  Standard errors are reported in parentheses.       -  Superscripts***, **and *indicate statistical significance of 1%, 5% and 10% level, respectively.

**Table 2.** Logistic regression model II. Dependent variable: “Impact of COVID-19 on stakeholder engagement of energy research projects – *Not at all*”.

Explanatory variables		Prediction model
Category	Name	Coefficient
**Stakeholder groups engaged**	Policymakers	-11.824 (6.787) *
	Civil Society Organizations	-9.133 (4.800) *
**Geographical/spatial focus**	Non-EU	-8.448 (5.098) *
**Importance of stakeholder engagement for the success of the project**	Not very important	25.546 (13.188) *
**Year of stakeholder engagement according to project plan/proposal**	2021	3.084 (2.814)
**Engagement activities according to the project plan/proposal (year 2020)**	Online survey	4.834 (4.365)
**Purpose of stakeholder engagement**	Access to data and information to understand a research problem	-3.660 (2.223) *
	Disseminate research results	10.459 (6.826)
**Priority of stakeholder engagement changed due to COVID-19**	Priority unchanged	4.116 (2.231) *
**Change in relationship to stakeholders**	No change	-8.377 (5.058) *
**Impact of changes in your stakeholder engagement activities on proceedings and results of your overall project**	Overall workflow is not impacted	4.882 (2.910) *
	No negative impact	4.328 (3.172)
	The project will be carried out as planned, with the envisioned results	6.973 (3.686) *
**Constant**		-17.752 (9.453) *

**Notes:**       -  Standard errors are reported in parentheses.       -  Superscripts***, **and *indicate statistical significance of 1%, 5% and 10% level, respectively.

**Figure 5.**
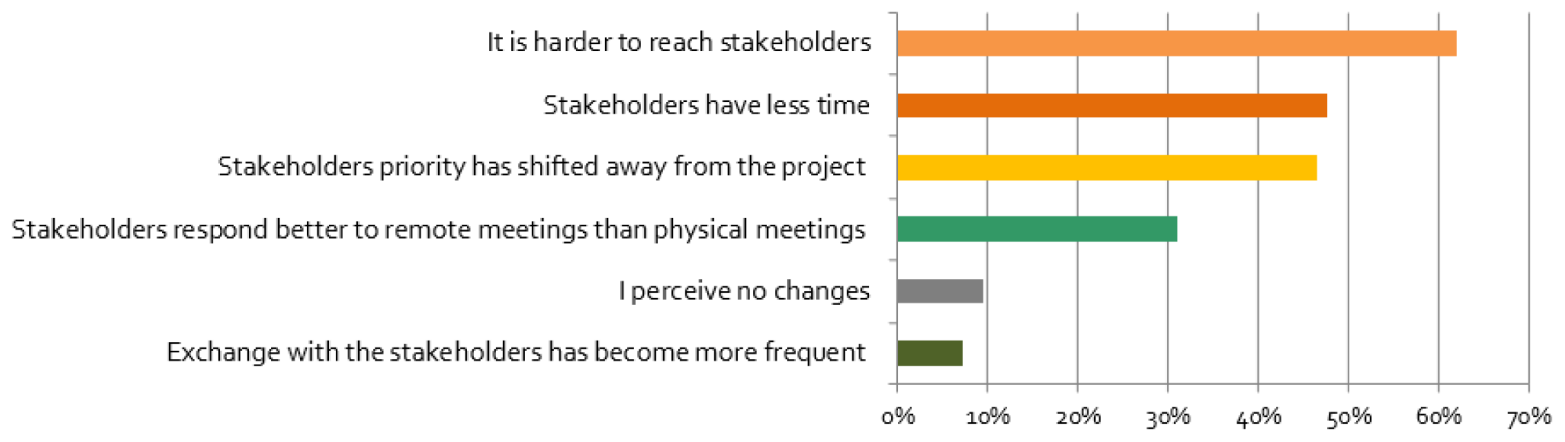
Perceived changes of relationship with stakeholders (multiple responses possible): *Please tick the boxes if you agree with the following statements*, n = 84.

Only 10% of the respondents reported that the COVID-19 measures had not effect on the stakeholder engagement ( Figure 4). For these projects, as expected, we find that if the interaction with stakeholders is ‘not very important’ for the project, the effect of the containment measures is smaller (cf. Table 2). Additionally, a small percentage assessed the influence of the pandemic to be positive ( Figure 4), which is possibly related to the better response of stakeholders to online formats ( Figure 5), enabling more frequent exchange with stakeholders or access to stakeholders living further away.

In contrast, for many respondents it became harder to reach stakeholders ( Figure 5). Respondents underlined that stakeholders’ life was negatively affected by the crisis: stakeholders *“experienced an increase in stress and workload”,* had *“difficult[ies] to perform the work foreseen due to the closure or reduction of activities*”, experienced dropped incomes, were *“unable to work”*, or may even have become unemployed. It is not surprising that these impacts have led to a shifting of stakeholders’ priorities away from the projects ( Figure 5). This result is also supported by our regression analysis, which indicated a positive correlation between negative effects and responses that stated that priority of engagement decreased very much since the beginning of the pandemic, and a positive correlation between no influence and responses that stated that priority of engagement was not affected at all. In contrast, the priorities for stakeholder engagement of most researchers did not change, but for some they decreased or increased. Respondents that were personally more affected are especially more likely to report that their stakeholder engagement priority has changed ‘very much’ (cf. Table 1). This indicates that people’s private life situation has an impact on their work life: with offices moving into homes, work and private life became linked more closely.

**Figure 6.**
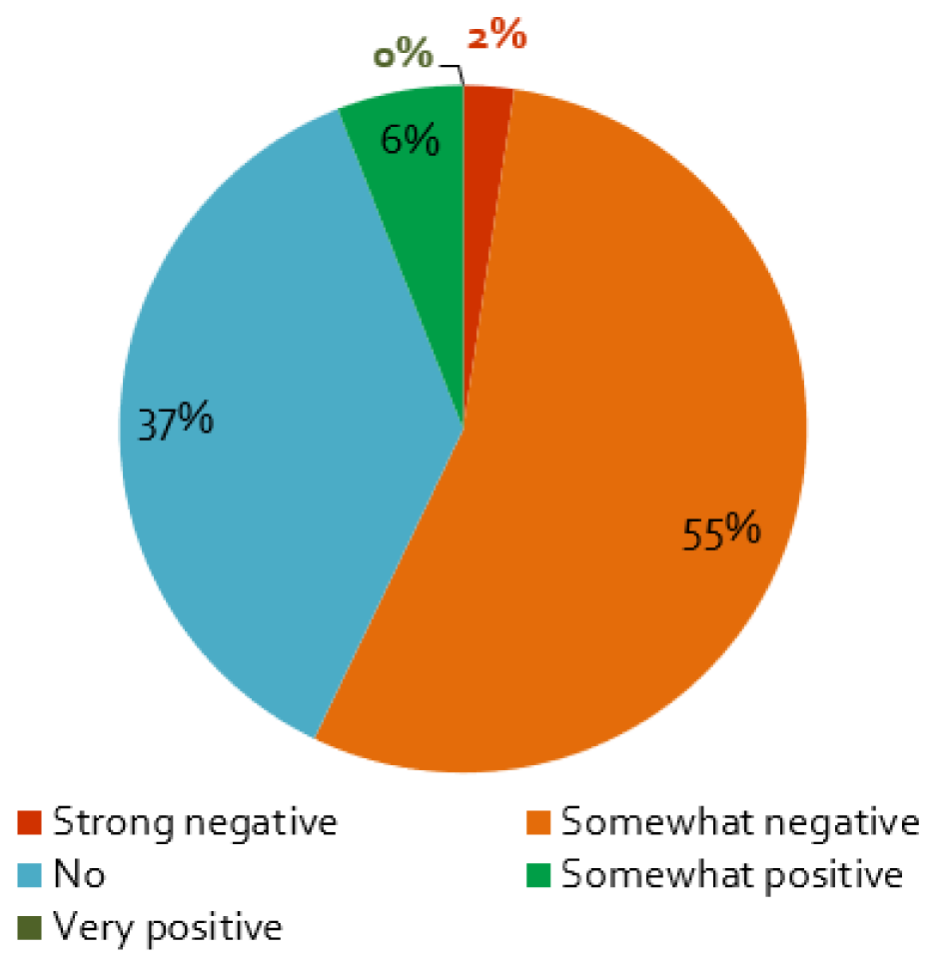
Impact of the coronavirus disease 2019 (COVID-19) pandemic on outcomes of the stakeholder engagement process: *Do you think that the COVID-19 situation will influence the outcomes of your stakeholder engagement process?*, n = 84.

More than half of the researchers expect a negative influence on the outcomes of the overall stakeholder engagement process ( Figure 6), not only in terms of engagement frequency and similar quantitative aspects, but also in the quality of interactions and stakeholder-based input for the projects. One responded explained:

*I think the COVID-19 restrictions on in-person community engagement will limit the value of stakeholder feedback. We have just completed the Comprehensive Plan update, and the four community engagement workshops created a bonding among the community that had great value. The participants felt ownership to the results and support the implementation actions. This attribute will be even more critical in the Climate Action Plan, as some of the actions are a bit more controversial. Lacking the interactive discussions and bonding over shared outcome is a weakness of the COVID-imposed process.*

This quote underlines that the more co-creative processes may also suffer from reduced possibilities for co-designing research questions, co-owning the results and co-agreeing on its implications – the very aim of transdisciplinary research.

### The impact of the COVID-19 crisis on project workflows and outcomes

**Figure 7.**
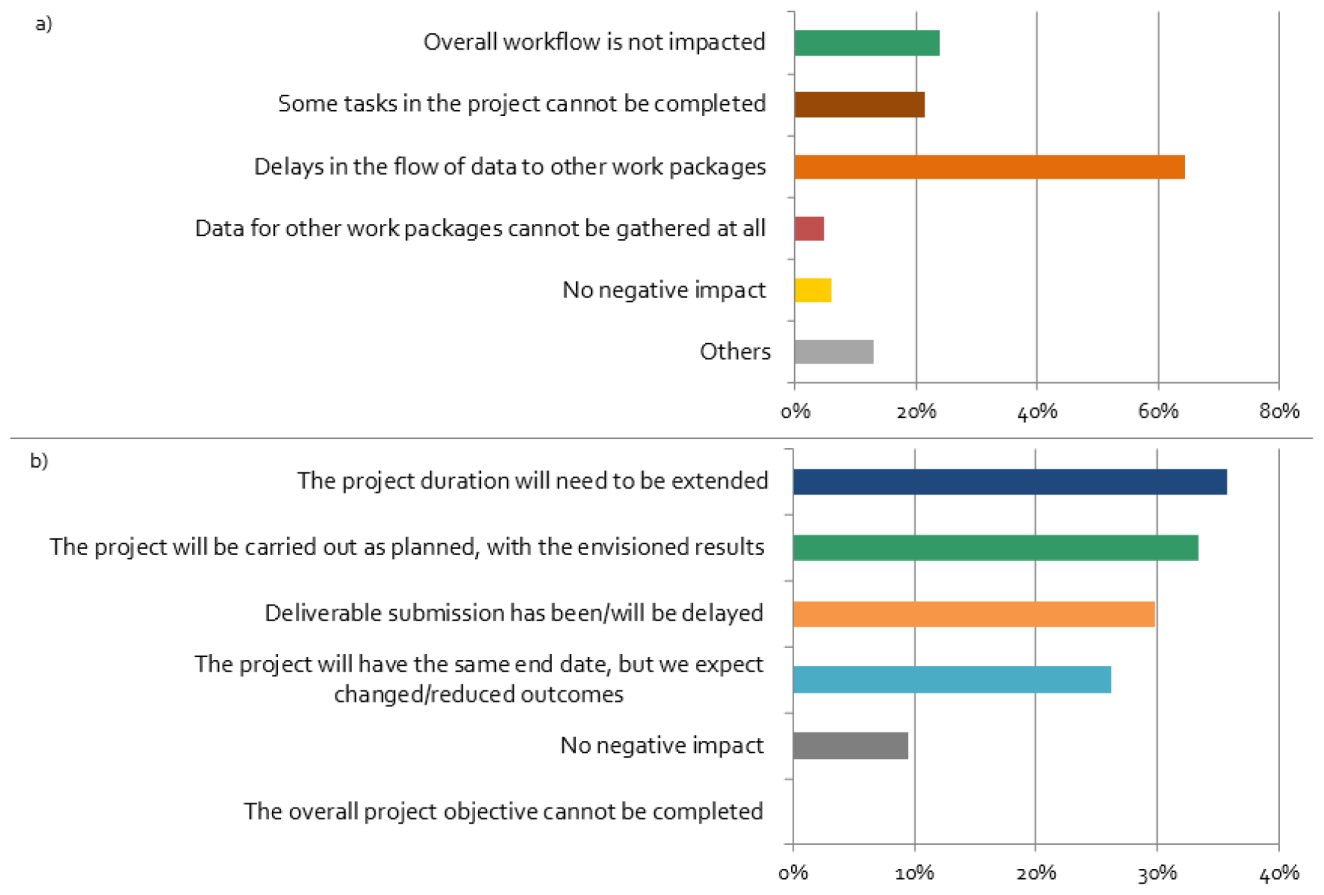
Impacts of the coronavirus disease 2019 (COVID-19) crisis on the projects: **a**) *Do you think that the COVID-19 situation will have a negative impact on the workflow within your project?*; **b**) *How do you think the changes in your stakeholder engagement activities will affect the proceedings and results of your overall project?* (multiple choices possible), n = 84.

The coronavirus restrictions have negative impacts on the workflow of most projects, mainly leading to delays in the flow of data between work packages, as shown in Figure 7a. We find a strong correlation between negative effects and the responses “Delays in the flow of data to other work packages”, “Deliverables’ submission has been/will be delayed” ( Table 1). This implies that delays have led to a stronger feeling by researchers’ that their stakeholder activities are negatively affected by the pandemic. This might be related to the fact that delayed input stemming from the stakeholder engagement process can be potentially considered in the project differently as initially planned.

Most respondents expected that changes in stakeholder engagement activities will affect the proceedings and results of the overall project ( Figure 7b). Although all respondents believe that the overall project objectives can be still completed, the majority of the projects will not be carried out as planned, and, thus, results will be different than expected. Furthermore, more than one third of the respondents expect that they will need to extend the project duration, which leads to their perception of stakeholder activities being ‘very negatively’ affected by the crisis (cf. Table 1). This is not only related to the challenge of involving stakeholders, but also because researchers had to *“adapt […] to this format[s] and approach[es that] require[d] a learning curve for [their] teams”.* This capacity building for dealing with online communication tools has been an important step for most of the respondents as few activities took place as physical, socially distanced events ( Table 3).

**Table 3.** Overview of engagement activities and impacts of the coronavirus disease 2019 (COVID-19) crisis on the implementation (numbers), multiple choices possible, n = 84.

Type of activity Alternative activities:	Planned	Implemented as planned	Socially distanced	Delayed	Cancelled	Format changed	Other
Originally planned:							
**Information events** **for stakeholders**	46	0	7	24	11	27	2
**Face-to-face** ** workshops**	64	0	9	29	10	45	7
**Conferences**	39	1	2	20	10	28	4
**Focus groups**	24	1	5	17	5	19	1
**Face-to-face** ** interviews**	28	1	1	14	6	16	2
**Online interviews**	14	8	-	8	0	3	1
**Face-to-face survey**	9	0	1	1	6	4	0
**Online survey**	24	18	-	7	1	0	0
**Webinars**	29	21	-	5	1	3	3
**% of strategy applied:**		18%	9%	45%	18%	52%	7%

### Coping strategies of researchers to deal with containment measures

Researchers adapted their involvement activities to the restrictions: only one of six stakeholder engagement activities were implemented as planned – almost all of which were already planned to be online – whereas two thirds were either cancelled or delayed. The most common coping strategies were the postponement of concrete stakeholder involvement activities, presumably hoping for looser restrictions in the future, and changes in formats – and often a combination of the two measures ( Table 3).

Among the alternative engagement formats (if formats were changed), online workshops and webinars were the most common ( Table 4). Conferences, face-to-face interviews and focus groups were often directly replaced by the respective online format. Information events, as well as face-to-face workshops, were mainly replaced by webinars and online workshops. Interestingly, respondents often performed more than one alternative engagement activity, suggesting that the online formats are not seen as perfect complements to physical meetings.

**Table 4.** Overview of alternative online engagement activities performed, if ‘format changed’ (numbers), multiple choices possible, n = 84.

Type of activity	Webinar	Online workshop	Online conference	Online focus groups	Online interviews	Online survey	Other
**Information** **events**	15	14	6	5	2	3	1 Online content, e.g., videos
**Face-to-face workshops**	17	32	5	7	6	3	5 Mailed survey, online group, not decided
**Conference**	10	7	18	1	1	2	4 Blogs, not decided
**Focus groups**	2	6	1	11	6	4	2 Not decided
**Face-to-face interviews**	1	1	0	1	14	1	2 Online meetings, telephone survey
**Face-to-face survey**	1	1	0	0	2	2	0
**Online interviews**	3	1	0	1	0	0	0
**Webinar(s)**	2	0	0	0	0	0	1 Not decided

### Assessment of alternative stakeholder engagement formats

**Figure 8.**
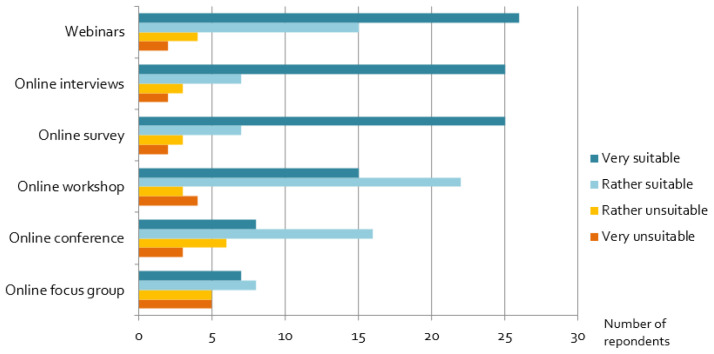
Assessment of alternative, online engagement methods: *Considering the goals of the stakeholder activity you wanted to perform originally, how suitable were the following formats as a replacement?*

Our results show that many alternative online formats – although not the researchers’ first choice − have been useful for projects: in particular, webinars, online interviews and online surveys are widely seen as suitable online engagement formats ( Figure 8). In contrast, experiences with online focus groups, online conferences and workshops were rather mixed. Interactive workshops and networking formats seem to be challenging, and respondents recommended rather short online conferences, because as one wrote *“online concentration span and endurance of people is limited*”. However, shorter events may lead *“sometimes to very superficial results because [there is] no time to deepen certain aspects”,* as one researcher reported. In addition, respondents suggested the splitting up of participants in *“smaller targeted online events (workshop, focus group, interviews), where not too many people are present”,* as well as *“break-out groups coupled with interactive polling tools, appeared to increase stakeholder retention and participation over the course of a small 1-day workshop.”*

For some projects it became easier to engage stakeholders online, especially “ *policymakers appear to be easier to engage in short online meetings than longer physical meetings”.* In contrast, other stakeholders left the projects, as consequence to the social distancing measures, as one respondent explained:

*Not all stakeholders […] wanted to continue meeting online. Many stakeholders found the planned in-person meeting of their peers in another city as a motivation to join the project in the first place.*

In addition, one quarter of the respondents stated that they could not reach stakeholders via digital tools, which concerned mainly citizens, local authorities, and locally-based businesses. One respondent expressed their concern: *“Many of our target stakeholders are elderly, and many have limited computer access.”*

Nevertheless, the majority (55 out of 66 respondents, free text reply) plans to continue online engagement activities after the contact restrictions are lifted; some were unsure about it yet, while a few expressed their scepticism – *“hopefully not”*. One respondent stated: *“The experience with the online workshop was good, so we will likely consider doing it again. However, this will not really replace face-to-face events. This is rather a complement to face-to-face workshops/activities”.* In their free-text responses, many respondents gave similar statements, agreeing that online formats cannot replace face-to-face meetings, as they are *“essential to engage with stakeholders to allow for clearer communication and networking”*.

## Discussion

### Impacts of the COVID-19 pandemic on stakeholder engagement activities

The COVID-19 crisis has affected stakeholder engagement in energy research mainly negatively, but it did not stop it. Adding to Leal Filho *et al*. (2020), we find that the pandemic has rearranged the work environment and the private life not only of energy researchers but also of stakeholders. Thus, it is not surprising that planning of, and participation in, engagement activities has moved down on professional agendas. Researchers reported a diversity of motives for engaging with stakeholders: while some mainly communicate results, others co-produce and co-design research and practical projects together. Projects that rely more on the involvement of stakeholders were more affected by the impacts of the pandemic.

Researchers coped with the pandemic most commonly by postponing activities and/or changing formats. Almost half of the projects decided to shift stakeholder engagement activities in time, likely in the hope that in-person communication will become possible soon. While this may initially be a useful strategy, to benefit from a deeper in-person engagement later on, delays may accumulate within a project. If tasks and workflows are interrupted, projects might fail to take stakeholder perspectives into account and/ or to finish projects on time. Furthermore, almost all engagement activities that took place have been moved online, which appears to be the most natural solution with social distancing measures in place. However, this also raises the question of how suitable the various digital formats are for engaging with stakeholders.

Researchers have had good experiences with some digital formats, but not with all of them. Webinars and online interviews, which are based either on one-to-one interaction or unidirectional communication, are found to work well online and provide satisfying results. Offering such formats online has clear advantages: it makes them easily accessible to a potentially larger audience of stakeholders, saves travel times and emissions. Moreover, they allow, for example, the recording of such activities, which makes evaluation work easier after the event. Contrary to this, formats requiring group activities, a higher level of stakeholder involvement and multidirectional, interactive communication (cf. Späth & Scolobig, 2017) are evaluated much less favourably by respondents. Consequently, researchers must carefully think about the objectives of the engagement and assess the suitability of online formats carefully: not all interaction types are equally suited for online formats. It also suggests that researchers could specifically continue using online formats in the future for one-to-one and unidirectional interactions with stakeholders, simply because they provide good results.

### Perspectives for stakeholder engagement after COVID-19

The COVID-19 crisis may prove to be a window of opportunity for digitalisation in stakeholder-involving research. We find that social distancing measures enforced an unforeseen shift to online engagement activities in five of six cases. This verifies findings by Schwarz *et al*. (2020) for the academic context: researchers are willing to also use digital tools for science-stakeholder interactions. Researchers quickly built up new capacities to involve stakeholders online and showed a high openness in trying new engagement formats instead of resigning. This is also true for stakeholders: although some stakeholder’s engagement in research projects decreased, most of them showed openness for new online engagement activities to varying degrees, but generally at least satisfactorily, the online formats worked too. This offers a positive perspective for stakeholder engagement while containment measures are still in place, but also shows that digital formats are likely to be used also after the pandemic, simply because they have proven their usefulness for particular aims. Furthermore, it can also contribute to the further improvement and development of online engagement formats, including the software and the specific approaches developed by researchers.

Nevertheless, adding to previous insights for the academic context ( Schwarz *et al*., 2020), we find that online engagement activities seem unsuitable to replace physical interactions with stakeholders completely. This is because a relatively short duration of online events reduces the depth of interaction and the quality of the results. This suggests that some projects might miss important information via online events, and the outcomes of the online stakeholder engagement must thus be critically evaluated.

In addition, we find that online events may not be able to replace physical events, because of different levels of stakeholders’ commitment to contribute to research projects: participating in physical events requires more time and other resources than switching on the computer. Hence, there is a risk of reduced commitment to online-only engagement processes. This implies that researchers who choose online formats need to rethink offline formats when applying them online to ensure that stakeholder are encouraged to join and are activated in online meetings. A complete shift from offline to online for all purposes does not appear possible or useful.

Last, we agree with Beaunoyer *et al.* (2020) that not all stakeholders are used to online technologies, which may lead to technology-related inequalities and digital exclusion of particular stakeholder groups. Hence, researchers must be aware of the stakeholder groups that cannot be easily reached digitally and adapt their approaches accordingly. For example, policymakers could be generally better reached via digital tools. However, on the other hand, projects that involved policymakers were more affected by the containment measures, probably because policymakers had to shift their priorities due to coronavirus-related issues. Nevertheless, in the future, we expect a shift towards a combination of online and offline activities at different times as well as hybrid formats that combine both forms.

### Implications on the future funding of research projects

Whereas stakeholder involvement is a crucial component of many research projects funded today, the COVID-19 crisis revealed that this requirement has made these projects vulnerable to changes in stakeholder availability and accessibility. Thus, it raises questions concerning the resilience of transdisciplinary research: access to stakeholders can only partially be influenced by the researchers themselves, and to some extent, they are simply exposed to the risk of failing engagement activities due to external factors. Consequently, the COVID-19 crisis can also be seen as a resilience test for the participatory aspirations of the research funding bodies. While the European Commission’s response has been generally sympathetic in that context, not all researchers were met with open ears when requesting for a project extension due to coronavirus restrictions. This issue may need to be addressed by funders, especially because coronavirus restrictions seem to continue for a longer time. Researchers, stakeholders, and funding institutions need to recognise the current situation and must stay flexible with their approaches.

### Limitations and prospects for future research

Measures against the COVID-19 pandemic are constantly changing, and so are the framework conditions for stakeholder interactions. Situations that respondents experienced in spring 2020 during the first wave of the pandemic may not necessarily be the same in spring 2021 or 2022. Moreover, as coronavirus restrictions also vary between countries, there are different possibilities for stakeholder interactions in different places. In this research, we could account neither for specific phases of the pandemic, nor for situations in specific country-based contexts; as a result, future research could investigate these changes and differences over space and time.

Since many activities were ongoing at the time of the survey, we could only identify initial lessons on the coping strategies and the success of implemented measures. Although we received insights on the *expected* impacts of coping strategies on the project outcomes, since the stakeholder engagement processes are ongoing, the *actual* impacts will likely materialise towards the end of the projects. To this end, an updated version of the same survey in 2021 or 2022 would be interesting and relevant. Furthermore, in this context, it would be valuable to learn from transdisciplinary research and projects where stakeholder engagement plays a vital role in co-creation processes how the shift to the online formats has influenced the outcomes. Most probably, more interactions in the digital world have not only serious implications on the general social reality, but specifically for approaches to complex, real-world problems related to sustainability.

In addition, the regression analysis performed was meant to supplement the explanatory analysis of the descriptive statistics and provide preliminary indications on the potential causalities that could explain the negative impacts of COVID-19 on stakeholder engagement activities in energy research projects. Scientists could use our publicly available dataset to perform more robust econometric analyses (incl. best-fitting model information criteria, evaluation of parameter estimates using quasi standard errors, etc.), also accounting for limitations due to the small sample size and number of responses in some variables that could cause issues of multicollinearity between the independent variables and affect the predictive power of the statistical models.

Finally, only energy research projects were within the scope of this study, with a focus on Horizon 2020 projects. Thus, our results are only generalisable beyond energy, where comparable results have been derived from similar research in other disciplines. It would be relevant to see similar studies also in other specific fields, to potentially monitor and compare how different research areas respond and adapt their approaches, to enable cross-disciplinary learning and an exchange of experiences.

## Conclusions

The coronavirus social distancing and lockdown measures have had a mainly negative effect on stakeholder engagement in energy research projects, especially by interrupting the exchange between researchers and stakeholders, causing delays in the project workflow, and changing outcomes of stakeholder engagement processes. Given this difficult situation, researchers have generally been able to quickly adapt by finding new ways of engaging with stakeholders, switching especially to online workshops and webinars. This shows that digital engagement has become a new norm during COVID-19. Researchers have had positive experiences with several online engagement formats, in particular webinars, online surveys and interviews – but are much less content with workshops and conferences. This suggests that online formats work particularly well for one-to-one and unidirectional formats, whereas interactive and group activities are less suited for digital formats. While various digital participation formats offer new opportunities for involving stakeholders more frequently by reaching a larger audience and being less time-consuming, an important disadvantage is that some social groups are excluded from the process and derived results might be more superficial. Thus, online engagement will likely continue after the crisis, but only to complement and not to replace physical meetings in energy research. The long-term effects on energy research remain to be seen. However, given the large amount of hope put into postponing events, there is a clear risk that projects will not be able to finish on time or with the intended contents, depending on the duration of the coronavirus-related restrictions.

Although the COVID-19 crisis is a monumental challenge for all, including for us ourselves as researchers, positive changes can be triggered. When forced to adapt, researchers and stakeholders quickly started experimenting with new formats – and developed several solutions that were found useful and attractive. Quite possibly, after the pandemic ends, we will find that more of our work has moved into the online space – and we will know why personal contacts are irreplaceable, after all.

## Data availability

### Underlying data

The survey data underlying this study are not openly available to protect the anonymity of participating individuals and projects. Given that we asked for many details of the projects, like funding source, duration etc., we cannot guarantee anonymity. For further COVID-19 research and the replicability of the research in another study context, access to semi-anonymised data can be granted. For further information on the data please contact Diana Süsser ( diana.suesser@iass-potsdam.de).

### Extended data

ZENODO: Questionnaire related to on the impact of COVID-19 on stakeholder engagement in European energy research (Version 1). http://doi.org/10.5281/zenodo.4765630 ( Süsser *et al.*, 2021) 

This project contains the following extended data:

- questionnaire_impact covid-19 on stakeholder engagment_SENTINEL_2020.pdf

Data are available under the terms of the Creative Commons Attribution 4.0 International license (CC-BY 4.0).
