# Supplementary material for: COVID-19 vs. stakeholder engagement: the impact of coronavirus containment measures on stakeholder involvement in European energy research projects
Source: Open Res Eur. 2021 Oct 14;1:57. Originally published 2021 May 25. [Version 3] doi: 10.12688/openreseurope.13683.3 (PMC10446069; doi:10.12688/openreseurope.13683.3)
Supplement: Peer review history

# Peer review: Reviewer response for version 2

**DOI:** 10.21956/openreseurope.15198.r27706

**Published:** 2021-10-07

**Authors:** Michael J. Fell

**Affiliations:** 1 UCL Energy Institute, University College London, London, UK

I can confirm I’m happy with the author responses to the review.”

If applicable, is the statistical analysis and its interpretation appropriate?

Partly

Is the study design appropriate and is the work technically sound?

Partly

Is the work clearly and accurately presented and does it engage with the current literature?

Yes

Are the conclusions drawn adequately supported by the results?

Yes

Are sufficient details of methods and analysis provided to allow replication by others?

Partly

Are all the source data and materials underlying the results available?

Partly

Reviewer Expertise:

NA

I confirm that I have read this submission and believe that I have an appropriate level of expertise to confirm that it is of an acceptable scientific standard.

# Peer review: Reviewer response for version 2

**DOI:** 10.21956/openreseurope.15198.r27707

**Published:** 2021-09-30

**Authors:** Walter Leal Filho, Amanda Lange Salvia

**Affiliations:** 1 European School of Sustainability Science and Research, Hamburg University of Applied Sciences, Hamburg, Germany

The authors have addressed our comments.

If applicable, is the statistical analysis and its interpretation appropriate?

Yes

Is the study design appropriate and is the work technically sound?

Yes

Is the work clearly and accurately presented and does it engage with the current literature?

Yes

Are the conclusions drawn adequately supported by the results?

Yes

Are sufficient details of methods and analysis provided to allow replication by others?

Yes

Are all the source data and materials underlying the results available?

Yes

Reviewer Expertise:

NA

We confirm that we have read this submission and believe that we have an appropriate level of expertise to confirm that it is of an acceptable scientific standard.

# Peer review: Reviewer response for version 1

**DOI:** 10.21956/openreseurope.14757.r26979

**Published:** 2021-06-25

**Authors:** Michael J. Fell

**Affiliations:** 1 UCL Energy Institute, University College London, London, UK

This paper uses a survey of EU energy research projects to describe the effect that Covid-19 response measures have had on stakeholder engagement. We find the approach to be quite clearly described and appropriate to the research questions. The findings are clearly described, although some clarification is needed around the regression analysis. More consideration of the implications of the findings would be valuable. We recommend the piece should be indexed, subject to a number of revisions.

 It would be useful if the scope of the work could be made a bit more precise. In the introduction it states “we investigate the effects of the COVID-19 crisis on the stakeholder work in European energy research”. However, participant recruitment seems to have been mainly aimed at Horizon 2020 projects, and the response rate reported is a % of EU-funded energy research project. The final sample was mainly Horizon projects (albeit a fairly small proportion of those eligible), plus some others. More clarity here, and consideration of implications of this for generalisability of the conclusions, is necessary.

 We are not fully clear on how exactly the regression analysis was conducted. Firstly, we were unable to identify exactly which survey question was used to determine the dependent variable. Please could this be clarified? The nearest one we could find was “How does the COVID-19 pandemic influence the stakeholder/engagement in your project? Likert scale: very positively <-> very negatively”. If the response options for the dependent variable were in the form of a response scale, how were responses which were not “Not at all” or “Very negatively” treated? It would also be useful if the authors could expand on why they decided to use logistic regression, and why it had to be run twice. It wasn’t quite clear how discrete choice modelling, and mentioned at the end of the methods section, was employed. Ideally more would also be said on how particular variables were selected for inclusion (i.e. one over the other), during the process of “examining in parallel the correlation matrix to test for collinearity issues among the covariates, until no significant collinearities are observed”.

 The regression results show a large number of variables, but the article only engages superficially with these findings. It would be helpful if the regression results could be moved to the results section, and some of the key effects pulled out and described in the text. It would then be useful to see some discussion of the regression results – both in terms of limitations due to sample size, but also on the more interesting findings. For example (while significance level is low), there was a large negative effect size for policymakers and civil society organisations in the “Not at all” analysis – could be implications of this be considered? Generally, the discussion is quite light on what the implications of the findings could be – both for the projects which data was captured on and going forward. Are/will important viewpoints more likely to be missed, e.g. those less able to access digital avenues for engagement?

 Some more minor points:

- It could be useful to provide an indicative timeline of some of the main Covid-related restrictions in Europe. This will obviously vary by country, but some general info could be helpful in interpreting this work in future
  .
- “Researchers agreed that online formats cannot replace face-to-face meetings” – is this based on a quantitative result from the survey and, if so, could this be included? Or is it just inferred from the quote?

If applicable, is the statistical analysis and its interpretation appropriate?

Partly

Is the study design appropriate and is the work technically sound?

Partly

Is the work clearly and accurately presented and does it engage with the current literature?

Yes

Are the conclusions drawn adequately supported by the results?

Yes

Are sufficient details of methods and analysis provided to allow replication by others?

Partly

Are all the source data and materials underlying the results available?

Partly

Reviewer Expertise:

Quantitative and qualitative social science research in energy.

I confirm that I have read this submission and believe that I have an appropriate level of expertise to confirm that it is of an acceptable scientific standard, however I have significant reservations, as outlined above.

# Response

**Published:** 2021-08-30

**Authors:** Diana Süsser

**Affiliations:** Institute for Advanced Sustainability Studies e.V. (IASS), Germany

Response to Reviewer 2 **R2.0 This paper uses a survey of EU energy research projects to describe the effect that Covid-19 response measures have had on stakeholder engagement. We find the approach to be quite clearly described and appropriate to the research questions. The findings are clearly described, although some clarification is needed around the regression analysis. More consideration of the implications of the findings would be valuable. We recommend the piece should be indexed, subject to a number of revisions.**

 We are very glad to read that the reviewer appreciates the descriptions of our approach, research question, and findings. We have expanded the discussion to add further aspects about the implications of our findings, and we have added details about the regression analysis (also in response to comment 1.1 above).

**R2.1 It would be useful if the scope of the work could be made a bit more precise. In the introduction it states “we investigate the effects of the COVID-19 crisis on the stakeholder work in European energy research”. However, participant recruitment seems to have been mainly aimed at Horizon 2020 projects, and the response rate reported is a % of EU-funded energy research project. The final sample was mainly Horizon projects (albeit a fairly small proportion of those eligible), plus some others. More clarity here, and consideration of implications of this for generalisability of the conclusions, is necessary.**

 Thank you for pointing this out. In the scope of the study were both Horizon 2020 and non-Horizon 2020 projects, but we only sent direct emails to the former. We allowed other projects to participate in our survey and we asked the projects if they were Horizon projects or not. We revised the text to make this clearer in the introduction and method section. We believe that many of our findings are generalisable beyond energy research – and specifically in cases where similar results have been found in other research and comment on this in the revised Discussion.

**R2.2 We are not fully clear on how exactly the regression analysis was conducted. Firstly, we were unable to identify exactly which survey question was used to determine the dependent variable. Please could this be clarified? The nearest one we could find was “How does the COVID-19 pandemic influence the stakeholder/engagement in your project? Likert scale: very positively <-> very negatively”. If the response options for the dependent variable were in the form of a response scale, how were responses which were not “Not at all” or “Very negatively” treated? It would also be useful if the authors could expand on why they decided to use logistic regression, and why it had to be run twice. It wasn’t quite clear how discrete choice modelling, and mentioned at the end of the methods section, was employed. Ideally more would also be said on how particular variables were selected for inclusion (i.e. one over the other), during the process of “examining in parallel the correlation matrix to test for collinearity issues among the covariates, until no significant collinearities are observed”.**

 Thank you very much for these insightful comments/ suggestions. We made sure to elaborate more in the text on how the regression analysis was performed, so that it is more comprehensible to the reader. The main changes in response to this comment are found in the Methods section.

**R2.3 The regression results show a large number of variables, but the article only engages superficially with these findings. It would be helpful if the regression results could be moved to the results section, and some of the key effects pulled out and described in the text. It would then be useful to see some discussion of the regression results – both in terms of limitations due to sample size, but also on the more interesting findings. For example (while significance level is low), there was a large negative effect size for policymakers and civil society organisations in the “Not at all” analysis – could be implications of this be considered?**

 We thank the reviewer for pointing out this very important issue. We have added more results to the results section. The Tables have been moved to the Results section. We added limitations of our approach to the limitations section of the Discussion and suggested further implications for research.

**2.4 Generally, the discussion is quite light on what the implications of the findings could be – both for the projects which data was captured on and going forward. Are/will important viewpoints more likely to be missed, e.g. those less able to access digital avenues for engagement?**

 We agree with the reviewers and hence, we expanded our Discussion and now emphasise the implications of our research better.

**R2.5 Some more minor points:** **It could be useful to provide an indicative timeline of some of the main Covid-related restrictions in Europe. This will obviously vary by country, but some general info could be helpful in interpreting this work in future.**

 We think that because of the difference timeline in the countries and given out study context, a detailed timeline is not necessary – or possible to generate with a reasonable time effort. We agree with the spirit of the comment, however, and added additional information about the containment measures in place in general during the time our survey was conducted (see Method section).

**R2.6 “Researchers agreed that online formats cannot replace face-to-face meetings” – is this based on a quantitative result from the survey and, if so, could this be included? Or is it just inferred from the quote?**

 Thank you for this question. We have adapted the text in the Results section (‘ ***Assessment of alternative stakeholder engagement formats’)*** to make this clearer. We did not specifically ask this question, but we got this statement as answers to open questions we asked. We asked the participants the open questions: *“Do you plan to continue using any of the new formats after restrictions are lifted?  Please briefly outline which ones and why” *(100% response) and *“Please explain briefly in what sense COVID-19 will influence the results of your stakeholder engagement process” *(79% response). Based on the answers given, we find that the majority plans to continue using online formats, but mostly in combination with physical formats, for example, *“[We will continue using] online workshop, but they cannot fully replace physical meetings”; “The experience with the online workshop was good, so we will likely consider doing it again. However, this will not really replace face-to-face events. This is rather a complement to face-to-face workshops/activities”; “An online workshop CANNOT replace a face to face meeting, as not all senses are used.”*

# Peer review: Reviewer response for version 1

**DOI:** 10.21956/openreseurope.14757.r26978

**Published:** 2021-06-07

**Authors:** Walter Leal Filho, Amanda Lange Salvia

**Affiliations:** 1 European School of Sustainability Science and Research, Hamburg University of Applied Sciences, Hamburg, Germany

The paper aims at investigating the effects of the pandemic on energy research, particularly in European projects with planned stakeholder engagement. The rationale for the study is well described and so are the discussions and implications.

 However, some improvements are needed as follows:

- The results/discussions could have been explored in a dedicated subsection or in more detail the contrasting results of the regression analysis, especially given the detailed methodology and the volume of information provided in the first tables.
- I recommend adjusting the title or adding a note to Table 4, to make it clearer for the reader e.g. explaining that the lines refer to the original/planned activities and that the columns refer to the alternative online ones.
- There are some light grammar/syntax errors on the text and it needs to be carefully edited. 
- Please provide/adjust the conclusions section in a way that you may summarise the main lessons from the paper, and outline future prospects.
- Each reference should be checked, to make sure they are all complete, with publisher, place of publication. Please check them all. Also, please cross check them as to make sure they are all on the text and in the references list. 

If applicable, is the statistical analysis and its interpretation appropriate?

Yes

Is the study design appropriate and is the work technically sound?

Yes

Is the work clearly and accurately presented and does it engage with the current literature?

Yes

Are the conclusions drawn adequately supported by the results?

Yes

Are sufficient details of methods and analysis provided to allow replication by others?

Yes

Are all the source data and materials underlying the results available?

Yes

Reviewer Expertise:

Sustainability and climate change

We confirm that we have read this submission and believe that we have an appropriate level of expertise to confirm that it is of an acceptable scientific standard, however we have significant reservations, as outlined above.

# Response

**Published:** 2021-08-30

**Authors:** Diana Süsser

**Affiliations:** Institute for Advanced Sustainability Studies e.V. (IASS), Germany

Response to Reviewer 1 **R1.0 The paper aims at investigating the effects of the pandemic on energy research, particularly in European projects with planned stakeholder engagement. The rationale for the study is well described and so are the discussions and implications.**

 We are glad to read that the reviewer values our study description, discussions, and implications.

**However, some improvements are needed as follows:** **R1.1 The results/discussions could have been explored in a dedicated subsection or in more detail the contrasting results of the regression analysis, especially given the detailed methodology and the volume of information provided in the first tables.**

 Thank you very much for this suggestion. We have enriched the results section with some more regression analysis-relevant explanations.

**R1.2 I recommend adjusting the title or adding a note to Table 4, to make it clearer for the reader e.g. explaining that the lines refer to the original/planned activities and that the columns refer to the alternative online ones.**

 We have adapted the line/column headings in Table 4 to make this clear.

**R1.3 There are some light grammar/syntax errors on the text and it needs to be carefully edited.**

 We have reviewed and proof-read the text again and corrected errors.

**R1.4 Please provide/adjust the conclusions section in a way that you may summarise the main lessons from the paper, and outline future prospects.**

 We have adapted the conclusion section. We now do not only summarise the main findings, but also provide some key implications. Furthermore, we outline prospects for future research to assess the more long-term impacts of the pandemic on stakeholder engagement and to identify best practices for stakeholder engagement beyond COVID-19.

**R1.5 Each reference should be checked, to make sure they are all complete, with publisher, place of publication. Please check them all. Also, please cross check them as to make sure they are all on the text and in the references list.**

 Thank you very much for this important note. We have cross-checked all the references and adapted the list accordingly.
